# Gene Expression in Developmental Stages of *Schistosoma japonicum* Provides Further Insight into the Importance of the Schistosome Insulin-Like Peptide

**DOI:** 10.3390/ijms20071565

**Published:** 2019-03-28

**Authors:** Xiaofeng Du, Malcolm K. Jones, Sujeevi S. K. Nawaratna, Shiwanthi Ranasinghe, Chunrong Xiong, Pengfei Cai, Donald P. McManus, Hong You

**Affiliations:** 1 Molecular Parasitology Laboratory, QIMR Berghofer Medical Research Institute, Queensland 4006, Australia; Xiaofeng.Du@qimrberghofer.edu.au (X.D.); s.nawaratna@griffith.edu.au (S.S.K.N.); Shiwanthi.Ranasinghe@qimrberghofer.edu.au (S.R.); Pengfei.Cai@qimrberghofer.edu.au (P.C.); 2 School of Veterinary Science, The University of Queensland, Gatton 4343, Australia; m.jones@uq.edu.au; 3 School of Medicine, Griffith University, Gold Coast 4222, Australia; 4 Key Laboratory of National Health and Family Planning Commission on Parasitic Disease Control and Prevention, Jiangsu Provincial Key Laboratory on Parasite and Vector Control Technology, Jiangsu Institute of Parasitic Diseases, Wuxi 214000, China; crxiong@hotmail.com

**Keywords:** *Schistosoma japonicum*, insulin-like peptide, RNA inference, immunolocalization, diagnosis

## Abstract

We showed previously that the *Schistosoma japonicum* insulin-like peptide (SjILP) binds the worm insulin receptors, thereby, activating the parasite’s insulin pathway and emphasizing its important role in regulating uptake of glucose, a nutrient essential for parasite survival. Here we show that SjILP is differentially expressed in the schistosome life cycle and is especially highly transcribed in eggs, miracidia, and adult female worms. RNA inference was employed to knockdown SjILP in adults in vitro, with suppression confirmed by significantly reduced protein production, declined adenosine diphosphate levels, and reduction in glucose consumption. Immunolocalization showed that SjILP is located to lateral gland cells of mature intra-ovular miracidia in the schistosome egg, and is distributed on the ciliated epithelium and internal cell masses of newly transformed miracidia. In schistosomula, SjILP is present on the tegument in two antero-lateral points, indicating highly polarized expression during cercarial transformation. Analysis of serum from *S. japonicum*-infected mice by ELISA using a recombinant form of SjILP as an antigen revealed IgG immunoreactivity to this molecule at 7 weeks post-infection indicating it is likely secreted from mature eggs into the host circulation. These findings provide further insights on ILP function in schistosomes and its essential roles in parasite survival and growth in different development stages.

## 1. Introduction

Schistosomiasis remains one of the most prevalent, insidious, and serious tropical parasitic diseases, with an estimated 200 million people infected in 76 countries [1,2]. There is no effective vaccine available currently [3], and treatment is entirely dependent on a single drug, praziquantel (PZQ); this is an ongoing concern because if PZQ-resistant schistosomes were to arise in the future this would pose a significant public health threat. Improved interventions for the control of schistosomiasis will rely on a better understanding of how schistosomes absorb host nutrients, neuro-endocrine hormones for their growth, development, and fecundity. It is well known that adult schistosomes consume their dry weight of glucose from host blood every 5 h [4] as this sugar is the major nutrient source for worm survival, growth, and maturity, and is required to meet the considerable energy requirements of mature female schistosomes especially which lay large numbers of eggs daily. Whereas there is much information regarding the mechanism of insulin signaling in *Caenorhabditis elegans* [5], it remains to be established whether the pathway and the regulation of glucose are similar in schistosomes. Genome-wide interrogation has revealed the presence of an insulin signaling pathway in *Schistosoma japonicum* [6], *Schistosoma mansoni* [7], and *Schistosoma haematobium* [8,9]. A total of 43 genes involved in the insulin pathway have been identified to date for *S. mansoni* [7], *S. haematobium* [9], and *S. japonicum* [6], including those encoding Src homology-containing (SHC), Src homology 2-B proteins, phosphoinositide-3-kinase (PI3K), extracellular signal-regulated kinase (ERK), glycogen synthase (GYS), and glucose transport protein 4 (GTP4).

The insulin-like peptide (ILP) and two types of insulin receptors (IR1 and 2), members of the large class of receptor tyrosine kinases, have been isolated from *S. mansoni* (SmILP [10] and SmIR1 and 2 [11]) and *S. japonicum* (*Schistosoma japonicum* insulin-like peptide (SjILP) [10] and SjIR1and 2 [12]). Our previous work with *S. japonicum* has shown that, by sharing the same binding epitopes [13], SjILP has a stronger binding affinity with the SjIRs than human insulin. However, the binding between the SjIRs and SjILP or between the SjIRs and host insulin can stimulate the schistosome insulin pathway and activate the downstream extracellular signal-regulated kinase (Erk)/mitogen-activated protein kinases (MAPK) and the serine/threonine kinase Akt (also known as protein kinase B)/phosphoinositide-3-kinase (PI3K) sub-pathways [10,14], both of which play an essential role in glucose uptake and growth, development, and fecundity in the adult worms [15]. We have demonstrated that SjIR1 is located on the surface of the adult schistosomes and is considered to play an important role in regulating the transport of glucose from host blood into the worms, a process triggered by its binding to host insulin or SjILP co-located on the tegument [10,12]. We hypothesize that the glucose taken up at the surface of worms is transferred into different parasite cells and tissues and that this process is activated by the binding between SjILP and SjIR-2. Both these components are co-located in the parenchyma of males and in the vitelline cells of the female vitellaria, where they play critical roles in the regulation of growth, adult fertility, and the differentiation of germline stem cell populations [10,13,14,16]. Compared with the inner tissue distribution of SjIR2 the location of SjIR1 on the tegument of adult *S. japonicum* suggests it may have direct access to host insulin which it is, thus, able to exploit. This is especially the case if SjILP is accidentally disrupted when host insulin can act as an emergency source to maintain the process of insulin signaling, thereby, enabling the schistosome to continue to take up glucose from the host. Furthermore, we showed previously that disruption of the insulin pathway in schistosomes by an antibody-mediated blockade of the binding between SjILP/human insulin and the SjIRs resulted in reduced glucose uptake and the starving and stunting of worms with subsequent retardation of sexual maturation and a reduction in egg output [12,13]. These outcomes were supported by the results of vaccine-challenge trials in mice using fusion proteins of the L1 subdomains (insulin binding domains) of SjIR1 and 2 (SjLD1 and 2) [13,17]. In murine vaccine/challenge experiments, we found that recombinant SjLD1 and 2 proteins induced a significant reduction of 56 to 67% in fecal eggs, a 75% reduction in mature intestinal eggs, the stunting of adult worms (~42% reduction in worm length), and a reduction in liver granuloma density [13,17]. These studies highlighted the important role in worm maturation and fecundity played by the schistosome insulin pathway, which needs to be activated by the binding between SjILP and SjIRs or between host insulin and SjIRs.

Despite these extensive findings, the biological function of SjILP has not been fully elucidated in the different life cycle stages of *S. japonicum*. In this study, we used real-time PCR to measure the transcription levels of SjILP in five different life cycle stages, and RNA interference (RNAi) was employed to explore the functional roles of SjILP in adult *S. japonicum*. Immunolocalization studies of SjILP were also undertaken in eggs, miracidia, and schistosomula. Furthermore, we determined the levels of SjILP-specific IgG antibodies by ELISA in serum samples collected from mice infected with *S. japonicum* at different time points in order to assess their potential value as a diagnostic marker of infection.

## 2. Results

### 2.1. Expression of SjILP in Different Developmental Stages of S. japonicum

**Figure 1:**
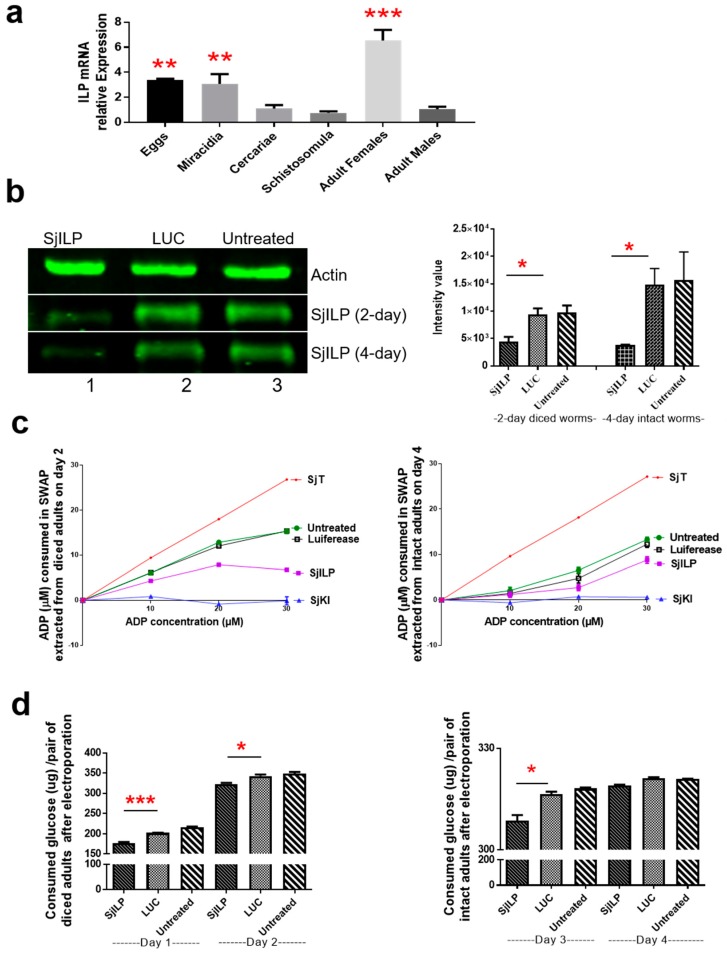
(**a**) Quantification of *SjILP* (*Schistosoma japonicum insulin-like peptide*) gene transcripts in different developmental stages of *S. japonicum*: eggs, miracidia, cercariae, schistosomula, adult female worms and adult male worms. *PSMD4* (26S proteasome non-ATPase regulatory subunit 4) was used as reference gene [20]. Data are representative of the mean ± SE of three independent experiments. *P* values were calculated using one-way ANOVA using the cercariae group as a comparison. (**b**) Western blot analysis of adult *S. japonicum* worm extracts obtained from diced and intact worms following treatment with SjILP dsRNA for 2 days and 4 days, respectively. Results are shown for proteins recognised by the anti-actin antibody (top panel) and anti-SjILP antibody (middle and bottom panel). Protein extracts in the middle panel were obtained from diced adult *S. japonicum* treated for 2 days with: dsSjILP (Lane 1); dsLUC (Lane 2); and without treatment (Lane 3). Protein extracts in the bottom panel were obtained from intact adults treated for 4 days. The intensity of anti-actin expression was evaluated so as to ensure equal protein loading. The experiment was repeated twice with similar results obtained. (**c**) Adenosine diphosphate (ADP) assays with soluble antigen preparation (SWAP) extracted from dsRNA SjILP/LUC treated/untreated adult *S. japonicum*. The ADP concentration (μM) was measured following incubation for 20 min with SWAP (0.13 mg/mL) obtained from dsRNA SjILP/LUC treated and untreated adult worms, a positive control (adult *S. japonicum* worm tegument protein extract; SjT) and a negative control (recombinant *S. japonicum* Kunitz type protease inhibitor (SjKI-1)). An ADP standard curve was obtained using different concentrations of ADP alone. This experiment was performed three times. (**d**) Glucose consumed by each pair of adult worms of *S. japonicum* after treatment with dsRNAs SjILP on days 1 and 2 in diced worms and on day 3 and 4 in intact worms. Data are representative of the mean ± SEM of three separate experiments. *P* values were calculated using *t*-test to compare the difference between SjILP RNAi group and the luciferase RNAi control group (* *p* value ≤ 0.05; ** *p* value ≤ 0.001; *** *p* value ≤ 0.0001). LUC, luciferase control group.

Real-time PCR was employed to quantify the gene transcript levels of SjILP in different life cycle stages (eggs, miracidia, cercariae, schistosomula, adult females, and adult males) of *S. japonicum*. SjILP was transcribed with the highest expression in adult female worms. Comparatively higher levels of SjILP transcripts were found in eggs and miracidia compared with schistosomula, cercariae, and adult male worms (Figure 1a).

### 2.2. Reduction in SjILP Protein Expression in Adult Parasites Treated with SjILP dsRNA

To determine whether the knockdown of SjILP dsRNA was reflected at the protein level, we performed Western blot analysis using soluble antigen preparation (SWAP) of diced adults harvested on day 2 post-treatment with dsRNA and intact adult worms obtained on day 4 post-treatment with dsRNA. Anti-SjILP antiserum and anti-actin antiserum (as a positive control) were used for the Western blot. SjILP were readily detected in an extract of control worms including luciferase dsRNA (dsLUC) treated worms and untreated worms. Markedly decreased levels of protein expression were evident in adult worms treated with SjILP dsRNA compared with the control worms (dsLUC treated or untreated worms) (Figure 1b). The levels of the control actin protein expression did not change in any of the test or control groups, demonstrating that comparable levels of protein were present in each lane (Figure 1b). Signal intensities of bands recognised by antibodies were measured using the Odyssey Classic Infrared Imager [18] with a scan intensity setting of 5 and sensitivity of 5. The intensities of the bands recognized by anti-ILP in the diced (2-day) and intact (4-day) parasites treated with SjILP dsRNA were reduced by 53% (*p* = 0.025) and 74% (*p* = 0.019), respectively, compared with control parasites (LUC) (Figure 1b).

#### 2.2.1. Adenosine Diphosphate Assays

We showed previously that SjILP possesses ADP hydrolyzing/binding activity [10]. This is noteworthy given adenosine diphosphate (ADP) plays a critical role in the inhibition of platelet aggregation and thrombus formation around the worms [19]. Here, we determined the ADP levels consumed in the SWAP of adult worms untreated or treated with SjILP dsRNA following their incubation for 20 min with different concentrations of ADP. After incubation with ADP, a negative control protein (recombinant SjKI) produced, as shown previously [13], a similar curve to that obtained with the ADP assay kit standard (Figure 1c). As a positive control, an adult *S. japonicum* tegument protein extract [13] hydrolyzed 88 to 94% of ADP after incubation with ADP (0–30 μM). There was no significant difference in the levels of consumed ADP between dsLUC treated and untreated worms. The SWAP isolated from 2-day diced worms treated with dsRNA SjILP and dsLUC consumed 22% and 51% of ADP (Figure 1c), respectively, whereas 4-day intact worms treated with dsRNA SjILP and dsLUC consumed 29% and 40% of ADP, respectively, following their incubation with 30 μM ADP for 20 min (Figure 1c).

The ADP hydrolyzing/binding ability of diced (2-day) and intact (4-day) adults treated with dsRNA SjILP was decreased by 56% (*p* ˂ 0.0001) and by 28% (*p* ˂ 0.0001), respectively, compared with the dsLUC knockdown control worms. These results indicated that 4-day intact worms, that had SjILP knocked down, had less ADP hydrolysis capability compared with 2-day diced worms (Figure 1c).

#### 2.2.2. Effect of SjILP Suppression on Glucose Uptake

Consumption of glucose from the culture medium by SjILP-suppressed adult worms was compared with that of LUC-knockdown and unsuppressed worm controls. In diced adult *S. japonicum*, the glucose consumed by each worm pair decreased significantly by 12% (*p ˂* 0.0001) and 6% (*p* = 0.01) on day 1 and day 2 after electroporation, respectively, in SjILP-suppressed worms compared with the LUC knockdown control group (Figure 1d). In intact worms, a decrease in glucose (2.5%, *p* = 0.01) consumed by each pair was observed in SjILP-suppressed worms on day 3, but not on day 4, post electroporation compared with the control group (Figure 1d). However, there was no difference in the levels of glucose consumed by dsLUC treated and untreated diced worms after 1 or 2 days incubation or by intact worms after 3 or 4 days incubation (Figure 1d).

### 2.3. Immunolocalization of SjILP in Eggs, Miracidia and Schistosomula

**Figure 2:**
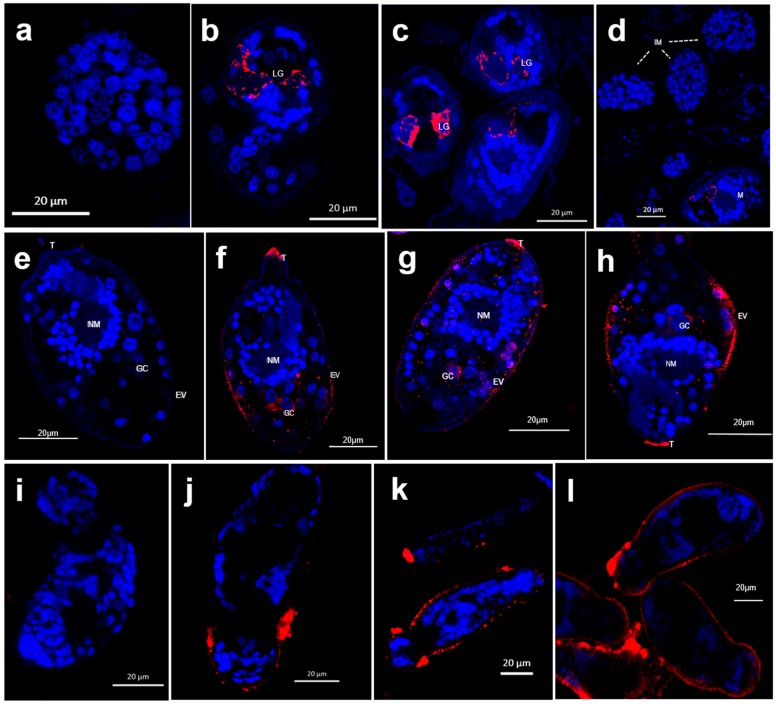
Immunofluorescence of *Schistosoma japonicum* insulin-like peptide (SjILP) in eggs, miracidia, and schistosomula following probing with mouse anti-SjILP antibody. Fixed mature eggs (**a**–**c**) and immature eggs (with one mature egg shown at the bottom of the panel) (**d**) isolated from livers of mice infected with *S. japonicum,* were incubated with: naïve control mouse serum (**a**) and mouse anti-rSjILP antiserum (**b**–**d**); fixed miracidia were exposed to naïve control mouse serum (**e**) and mouse anti-rSjILP antiserum (**f**–**h**); fixed schistosomula were exposed to naïve control mouse serum (**i**) and mouse anti-rSjILP antiserum (**j**–**l**). All samples were then incubated with Alexa fluor^®^ 647 goat anti-mouse IgG (red fluorescence). DAPI stained nuclei are blue. M-mature egg; IM-immature egg; LG-lateral gland cell; NM-neural mass; GC-germinal cell; EV-excretory vesicle; T-terebratorium.

Immunofluorescence of eggs showed that native SjILP was localized to lateral gland (LG) cells of the intra-ovular miracidium within mature eggs (Figure 2a–d), whereas no SjILP staining was observed in immature eggs (IM) (Figure 2d) [the single fluorescant mature egg (M) present at the bottom of the panel]. The newly hatched miracidia are pyriform (Figure 2e–h). SjILP is shown present both within the distal cytoplasm of the lateral ciliated epithelial plates of the miracidia and also internally in cell masses (Figure 2f–h), including germinal cells (GC) and apical papillae of the terebratorium (T). In day 1 schistosomula, focal densities of SjILP immunoreactive material was observed in the distal cytoplasm of the tegument in the antero-lateral region of the larval worm (Figure 2j–l). The dense regions of label are bound posteriorly by a less intense but positive, and in some individuals, punctate immunoreactivity along the tegument of the worms. This less intense label extends almost to the posterior regions of the parasites (Figure 2k,l).

### 2.4. Native SjILP in Soluble Egg Antigens and Egg Secreted Proteins Detected by Western Blot

**Figure 3:**
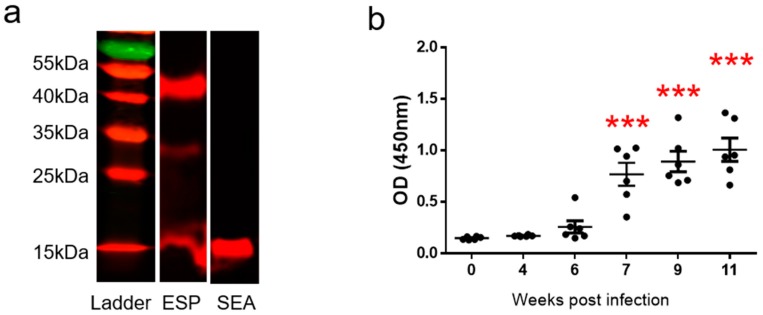
(**a**) Western blot analysis using mouse anti-SjILP serum to probe ESP (egg secreted proteins) and SEA (soluble egg antigens). (**b**) IgG antibody response against recombinant SjILP antigen in the sera of BALB/c (laboratory bred mouse strain of albino genotype c) mice at 0, 4, 6, 7, 9, and 11 weeks post-challenge infection with 16 *Schistosoma* *japonicum* cercariae. Statistical significance was determined using one-way ANOVA. (*** *p* < 0.0001).

To determine whether native SjILP is secreted by *S. japonicum* eggs, we undertook further Western blot analysis using mouse anti-SjILP to probe soluble egg antigens (SEA) and egg secreted proteins (ESP). Anti-SjILP antibody recognized SEA at the expected molecular size of 15 kDa (Figure 3a) and ESP at the predicted sizes of 15 kDa (monomeric form), 30 kDa (dimeric from), and 45 kDa (trimeric form) (Figure 3b). It has been shown that SjILP [10] is characterized by having a signal peptide and the six strictly conserved cysteines forming three disulfide bonds resulting in a functional structure of the dimeric or trimeric peptide [16]. However, this dimeric or trimeric native SjILP structure may be damaged during the preparation of SEA under denaturing conditions using lysis buffer containing urea.

### 2.5. Kinetics of Anti-SjILP IgG Antibody in the Sera of S. japonicum-Infected Mice

Serum samples from six infected mice were collected before infection (week 0) and at 4, 6, 7, 9, and 11 weeks post challenge infection with *S. japonicum* cercariae. Using ELISA we found that specific anti-SjILP IgG antibody levels were elevated during the course of the infection; there was a significant increase in antibody levels against SjILP detectable from 7 weeks post-infection (*p* < 0.0001) (Figure 3b).

## 3. Discussion

Previously we demonstrated the presence of SjILP and IRs in *S. japonicum*, that SjILP has a stronger bind affinity with the SjIRs than human insulin, and that the binding between the parasite IRs and parasite ILP/host insulin can activate the schistosome insulin pathway [10,12] which plays a critical role in adult worm growth, development, and fecundity [15]. In the current study, consistent expression levels of SjILP were observed in all the life cycle stages of *S. japonicum* examined indicating a critical role for SjILP in meeting the energy demands of the different developmental stages of this parasite. Transcription of SjILP was highly up-regulated in females, suggesting an important role in helping to regulate glucose uptake in these worms which demand considerable energy requirements to mature and lay massive numbers of eggs during the sexual reproductive phase of the life cycle of *S. japonicum*. The higher level of expression of SjILP in female *S. japonicum* than in males may imply that the latter may absorb more host insulin due to the large tegumental surface exposed to host blood, while females, held inside the gynaecophoric groove of males, have to depend more on their own ILP for growth and survival. This finding also substantiates our earlier published finding that unpaired female worms can take up glucose directly from the environment in the presence or absence of host insulin [14].

RNAi was employed to knock down SjILP in adult *S. japonicum*, and the efficacy of the knockdown was evaluated by measuring the levels of SjILP protein expression, ADP hydrolyzing/binding ability, and the amount of consumed glucose in the suppressed diced and intact adult worms. Previous studies showed that primers designed for qPCR to measure the knockdown of target mRNA should not flank the dsRNA target site [21] since the detection of the dsRNA itself can, in some cases, lead to hybridization with non-target gene sequences [22]. Given the fact that the full-length SjILP cDNA comprises an open reading frame of 390 bp and the dsRNA SjILP designed for RNAi covered 318 bp nucleotides (excluding the signal peptide of SjILP), we did not measure the transcription level of SjILP as a parameter to evaluate the knockdown efficacy. However, the significant suppression in SjILP protein level was observed in SjILP knockdown adult worms as shown by Western blot analysis (Figure 1b). An important function of the *S. japonicum* ILP is that it possesses ADP hydrolyzing/binding activity which is notable as ADP is a key metabolite in energy transfer reactions and, as indicated earlier, also plays a pivotal role in inhibiting platelet aggregation and thrombus formation around the worms [19]. The decline in ADP hydrolyzing/binding ability in diced adults (56%) on day 2 post-treatment with SjILP dsRNA and intact (28%) adults on day 4 compared with the LUC control worms (Figure 1c) reflected the knockdown efficacy. Increased inhibition of ADP hydrolyzing/binding activity was observed in fragmented adult parasites, an outcome supported by a previous report showing increased knockdown efficacy of RNAi with diced adult schistosomes [23]. However, to ensure the fragmented adults were alive at the time of collection for analysis, we harvested the worms on day 2 post-treatment and carefully checked them under the microscope. Intact adults were harvested on day 4 post-treatment to provide sufficient time for phenotypic changes to become apparent. However, increased suppression in the level of SjILP protein was observed on day 4 in intact *S. japonicum* (74%) compared with day 2-treated fragmented adults (53%) (Figure 1b), implying intact adults possess an integrated mechanism/system that can be used in response to the dsRNA treatment.

Another important effect of SjILP knockdown was a significant decline in the level of consumed glucose in diced worms on day 1 and 2 after dsRNA SjILP treatment and in intact worms on day 3 compared with control worms treated with LUC (Figure 1d). However, there was no difference in the level of consumed glucose between the SjILP dsRNA and LUC treated adults on day 4 post-treatment, when the expression of SjILP was shown by Western blot to be depressed by 74% compared with the control worms treated with LUC (Figure 1b). As we hypothesized, this may indicate a transient role for human insulin in regulating glucose uptake in *S. japonicum* when the expression of SjILP was depressed by RNAi, with the parasite utilizing host insulin to maintain its glucose metabolism at the level required for survival. This point is supported by our previous findings [10] that host insulin is present on the surface of adult *S. japonicum* after their perfusion from the mouse model followed by several washes with perfusion buffer. By sharing the same binding sites present in the SjIRs, host insulin exhibited 2.8 to 5.5 times less binding to SjIRs than to SjILP [10] indicating competition between the two molecules. Furthermore, knockdown adult *S. japonicum* were cultured in medium complemented with 20% of heat-inactivated fetal calf serum (FCS) which contains bovine insulin. Bovines are recognized natural reservoir hosts of schistosome infection, and bovine insulin has very similar physiological effects as human insulin [24] due to the highly conserved amino acid sequence of insulin among vertebrates (including human, bovines, and mice). Another explanation could be that the process of glucose uptake in schistosomes may be modulated or regulated by multiple genes outside of the central insulin signaling pathway. Previous studies have shown that glucose transport in schistosomes is also regulated by acetylcholine interaction with parasite acetylcholine receptors and acetylcholinesterase present in host blood [25,26]. Furthermore, interleukin-7 (IL-7) and thyroid hormone thyroxin (T4) are able to regulate schistosome glucose metabolism through modulations in the circulating levels of host glucose and insulin [27].

Whereas transcription of the SjIRs occurs only in the mammalian host stages (schistosomula, males and females) of *S. japonicum* [12], we show here that SjILP is also expressed in eggs, miracidia, and cercariae suggesting that, in addition to binding SjIRs and activating the parasite insulin pathway, this molecule is involved in other cellular functions in this parasite. The significantly higher levels of expression of SjILP in eggs and miracidia suggest that it plays an important role in the provision of sufficient energy for egg hatching or to sustain the rapid and sustained movement of miracidia in fresh water enabling them to find a suitable intermediate snail host in a limited time period. The relatively low expression of SjILP in the schistosomulum, the earliest developmental stage in the mammalian host after cercarial invasion, suggests that this immature schistosome may depend more on host insulin than SjILP to help satisfy its energy requirements for growth and development. This observation gains support from earlier work showing that the addition of human insulin to a chemically defined synthetic medium greatly increased the survival time and improved the viability of schistosomula in vitro [28].

Immunolocalization, using an anti-SjILP antibody, showed that SjILP was not detectable in immature eggs but that it was present at a high level in mature eggs, being especially localized to the pair of lateral glands of fully formed miracidia, which differentiate late in egg development (stages 6 and 7) [29] when glycoprotein production commences. This localization pattern suggests SjILP might be secreted by the lateral glands or is derived from an excretory system [30]. Western blot analysis with the anti-SjILP antibody showed that native SjILP was recognized, at the expected molecular size, in SEA and ESP, again indicating that SjILP is present in eggs and can be released/secreted from eggs into the mammalian host circulation. Furthermore, we tested for anti-SjILP IgG antibodies in the sera of laboratory mice infected with *S. japonicum* at different time points post-cercarial challenge. We found specific SjILP antibodies were first significantly detectable at 7 weeks post-infection when considerable numbers of mature *S. japonicum* eggs are being laid, implying that SjILP is secreted by mature eggs trapped in the liver or intestine. It is well recognized that *S. japonicum* females begin laying eggs 4 weeks after infection and that schistosome eggs are unembryonated (immature) when they are first laid and pass into the host circulation [31]. During embryonation over 1 week, the eggs develop extraembryonic envelopes beneath the shell. Of these, the inner envelope is highly active metabolically and is said to be the major source of the egg secretions [31]. The host granuloma response to schistosome eggs is strictly dependent on viable mature eggs and does not occur around single immature eggs [29]. It has been suggested that the secretion of egg proteins to periovular tissues may be related to the formation of structures that immediately underlie the shell during egg maturation [31]. The shell has numerous pores through which these secretions can be released into the host circulation. However, the precise role and function of SjILP in inducing the host immune response and subsequent granuloma formation remain to be determined.

Schistosome eggs hatch on contact with water, following their release from the mammalian host into the external environment. During hatching, water enters through pores in the egg into the extra-embryonic spaces and the resulting swelling (turgescence) causes a rupture in the egg shell, releasing the miracidium [29]. We show that SjILP is present both on the ciliated surface of the newly released miracidia and internally in cell masses (Figure 2f–h), including germinal cells, which are critical in the transformation of miracidia into mother sporocysts [32], suggesting that SjILP might also be involved in sporocyst growth and development [33]. Miracidial penetration into the schistosome snail intermediate host leads to the shedding of ciliary epidermal plates from the larval surface during the development of the new tegumental syncytium of the developing sporocyst [34]. Surface proteins (including SjILP) involved in the miracidium-to-sporocyst transformation [35] might also be important in helping to modulate the immune response in the snail intermediate host. Furthermore, immunolocalization showed SjILP staining was detected in schistosomula in large paired masses on the antero-lateral margin of the tegument laterally to the ventral sucker, and in discrete patches along the lateral margins of the tegument. The abundance of SjILP in the antero-lateral tegument would suggest that the molecule is produced in areas of rapid growth and differentiation in the early development of schistosomula. The high level of expression of SjILP around this region suggests a critical role for SjILP in the development of schistosomula into adult worms (Figure 2j–l). It is well recognized that parasite surface proteins, especially those expressed in/on the tegument of schistosomula, represent key targets as vaccine candidates and in the diagnosis of an early schistosome infection [36], given the critical role the tegument at the interface between host and parasite and the fact that tegumental molecules undertake functions essential for parasite survival. Furthermore, a recent study [16] has demonstrated the presence of insulin signaling pathways in the genomes of *Echinococcus granulosus*, *E. multilocularis*, *Hymenolepis microstoma*, *Taenia solium*, *T. saginata*, and *T. asiatic* and the participation of putative insulin-like peptides in cestode reproduction. These peptides exhibited 45 to 61% identity with SjILP/SmILP, sharing strictly conserved features of the insulin family suggesting similar roles in the growth, development and maturation of these tapeworms [16]. The identification of these insulin-like peptides improves our understanding of the insulin signaling pathways in the flatworm parasites, and on host–parasite interactions.

## 4. Materials and Methods

### 4.1. Ethics Statement

The conduct and procedures involving animal experimentation were approved by the Animal Ethics Committee of the QIMR Berghofer Medical Research Institute (project number A0108-054 ongoing from 1992 with annual update approvals).

### 4.2. Parasites

*Oncomelania hupensis hupensis*, infected with *S. japonicum*, were obtained from an endemic area in Anhui Province, China, and transported to the Brisbane laboratory in Australia. Cercariae were shed from the infected snails and collected as described [37].

### 4.3. Real-Time PCR to Measure the mRNA Expression Level of SjILP in Different Life Cycle Stages of S. japonicum

Total RNA was extracted separately from *S. japonicum* eggs, miracidia, cercariae, schistosomula, adult female and male worms as described [12]. First strand cDNA synthesized using a Sensiscript Reverse Transcription for First strand cDNA synthesis Kit (QIAGEN, Hilden, Germany), was subsequently used as template in qPCR to determine the expression level of SjILP. *PSMD4* (26S proteasome non-ATPase regulatory subunit 4) was used as the reference gene [20]. Forward (5′-AGTTCGTTTCAAAAGATCACTGGA-3′) and reverse (5′-AGATAACTTCGTGTGCATCCAA-3′) primers for SjILP were designed using the Primer 3 software (http://frodo.wi.mit.edu/), and the specificity of primer sequence was confirmed by Basic Local Alignment Search Tool (BLAST). Experiments were performed with QuantiNova SYBR Green PCR Kit (QIAGEN). Relative expression levels were normalized to *PSMD4* and calculated using the 2^−ΔΔ*C*t^ method [38].

### 4.4. Treatment of Parasites with Double Stranded RNA (dsRNA)

As we previously reported [39], freshly perfused adult *S. japonicum* worms were incubated in Dulbecco’s Modified Eagle Medium (DMEM) (Invitrogen, Carlsbad, CA, USA) medium, supplemented with 20% (*v*/*v*) heat-inactivated fetal calf serum (FCS), 100 IU/mL penicillin and 100 mg/mL streptomycin at 37 °C in an atmosphere of 5% CO_2_ in air overnight.

dsRNA targeting SjILP [18] (KX268651) was synthesized from *S. japonicum* cDNA using gene-targeted primers containing T7 promoter sequences:

F: 5′-TAATACGACTCACTATAGGAGATACAACACATAGTTTACCAGAATTACA-3′

R: 5′-GCAAAATTGTTCTAGATAACTTCGTAGAGGGATATCACTCAGCATAAT-3′ dsRNA was synthesized and purified using a Megascript RNAi kit (Ambion, Foster City, CA, USA). Luciferase dsRNA (LUC) was used as a negative control [39,40,41].

Given recent studies showing that dicing adult schistosomes for RNAi can result in more gene reporter activity than intact worms [23], we used diced and intact adult worms for the dsRNA treatment. Accordingly, adults of *S. japonicum* were removed from overnight culturing after perfusion and diced into three fragments using a sharp, sterile blade. Intact or fragmented adults were moved into a 0.4 cm cuvette (BIO-RAD, Hercules, CA, USA) and the medium was replaced with 50 µl fresh and cold Opti-MEM medium containing 25 ug dsRNA and subjected to square wave electroporation (125 V, 20 ms, one pulse). After electroporation, the worms were transferred into pre-warmed supplemented DMEM and incubated at 37 °C under 5% CO_2_ in air. The culture medium was collected (20 μL/well) and its glucose concentration measured using a glucose assay kit (Biocore, Gaithersburg, MD, USA). Adult fragments were harvested on day 2 after electroporation when they were still alive shown by checking under a microscope. Intact adult worms were collected on day 4. SWAPs of these fragmented and intact worms were prepared and subsequently used for Western-blot analysis and ADP activity assay as described below.

#### 4.4.1. ADP Assays

To measure the level of hydrolysis of ADP in SjILP knockdown adult schistosomes, ADP assay kits (Sigma-Aldrich, city, country) were used to measure ADP levels of SWAPs (0.13 mg/mL) extracted from dsRNA treated or untreated adult *S. japonicum* after incubation with ADP (0, 10, 20, 30 μM) for 30 min. These proteins included: SWAPs extracted from adult *S. japonicum* treated with (i) SjILP dsRNA; (ii) LUC dsRNA; (iii) untreated; and (iv) *S. japonicum* tegumental protein as a positive control [10,42], extracted from adult *S. japonicum* using the freeze/thaw/vortex method [43]; (v) *S. japonicum* Kunitz type protease inhibitor (rSjKI-1) [44] as a negative control, which was shown to have no ADP binding ability [10].

The amount of consumed ADP (µM) of worms incubated with 30 µM ADP = 30 (µM) − ADP maintained in SWAP (µM)

The reduction of ADP hydrolyzing/binding ability (%) = 100 × (Consumed ADP in worms treated with dsLUC—Consumed ADP in worms treated with dsRNA SjILP)/Consumed ADP in worms treated with dsLUC.

#### 4.4.2. Western-Blotting

(1) Detection of SjILP protein knockdown levels in adult worms and eggs after RNAi treatment.

Adult fragments harvested on day 2 post-treatment and intact worms collected on day 4 were lysed with 1% (*v*/*v*) Triton X-100 in Tris buffered saline supplemented with complete protease inhibitor cocktail (Sigma, St Louis, MO, USA) [12]. The prepared SWAP samples from dsRNA treated and untreated groups were separated on a 15% (*w*/*v*) sodium dodecyl sulfate- polyacrylamide gel electrophoresis (SDS-PAGE) gel and transferred to an Immun-Blot^®^ low fluorescence- Polyvinylidene difluoride (PVDF) membrane. Overnight blocking was performed with Odyssey buffer (Li-COR Biosciences, Lincoln, NE, USA.) containing 2% (*v*/*v*) goat serum at 4 °C. Then, the membrane was subjected to incubation with the mouse anti-SjILP anti-serum we generated previously [10] (1:100 dilution in Odyssey buffer and 0.1% Tween-20) for 1 h followed by incubation with IRDye-labeled 680LT goat anti-mouse IgG antibody (Li-COR Biosciences) (1:15,000 diluted in Odyssey buffer with 0.1% Tween-20 and 0.01% SDS) for 1 h. An anti-actin antibody (Sigma-Aldrich) (1:150) [45] was used to assess protein-loading differences. After a final wash with distilled water, the membrane was allowed to dry in the dark and visualized using the Odyssey^®^ CLx Infrared Imaging System [18].

(2) Recognition of SjILP protein in prepared *S. japonicum* soluble egg antigens (SEA) and egg secreted proteins (ESP).

SEA and ESP were prepared as described [46]. Briefly, SEA purified eggs were homogenized in 50 mM Tris HCl (pH 8.8) buffer [containing 7 M urea, 2 M thiourea, 4% 3-[(3-cholamidopropyl) dimethylammonio]-1-propanesulphonate (CHAPS), protease inhibitor cocktail (all Sigma)] on ice, followed by centrifugation at 25,000 g for 1 h at 20 °C. ESP was collected by incubating mature eggs in serum-free RPMI (Invitrogen) for two periods of 3 days with a medium change interposed. The mouse anti-SjILP anti-serum [10] was used to probe to the native SjILP protein in an SDS-PAGE separated SEA and ESP as described above.

### 4.5. Immunolocalization of SjILP in Eggs, Miracidia and Schistosomula

Eggs were isolated and purified from the livers of mice infected with *S. japonicum* as described [47]. Samples enriched for immature or mature eggs were produced as reported [31] followed by fixing in 10% (*w*/*v*) formalin in PBS. The mature eggs were also hatched in deionized water under light as described [47], and the miracidia were harvested by centrifuging at 500 g for 5 min followed by fixing in 10% (*w*/*v*) formalin in PBS. *S. japonicum* cercariae were obtained by shedding infected *Oncomelania hupensis hupensis* under a bright light. Cercariae were transformed mechanically to schistosomula [48] and cultured in Basch’s medium [49] overnight. The one-day-old schistosomula were fixed in 10% (*w*/*v*) formalin in PBS. Revealt A solution (Biocare Medical, Concord, MA, USA) was added to the fixed eggs, miracidia and schistosomula and the parasite stages heated at 120 °C for 5 min for antigen retrieval. This was followed by washing 3 times with 0.05% (*v*/*v*) Tween 20 in Tris buffered saline (TBS-T) and the parasites were then blocked with 1% (*w*/*v*) bovine serum albumin and 1% (*w*/*v*) goat serum in Tris buffered saline (TBS) as described [26] for 60 min, and then incubated with mouse anti-SjILP antibody (1:200) at 4 °C overnight. After three washes with TBS-T, samples were incubated with Alexa fluor^®^ 647 goat anti-mouse IgG (1:500) (Invitrogen, Carlsbad, CA, USA) at 37 °C for 1 h. Nuclei in the tissue sections were counterstained with diamidino-2-phenylindole (DAPI) gold (Invitrogen, Carlsbad, CA, USA) and visualized under fluorescence using a Zeiss 780 NLO confocal microscope (Zeiss, Oberkochen, Germany).

### 4.6. Kinetics of Anti-SjILP IgG Antibody Levels in the Sera of S. japonicum-Infected Mice Determined by ELISA

Eight-week-old BALB/c mice were percutaneously infected with 16 *S. japonicum* cercariae. Blood samples for sera were taken from animals at 0, 4, 6, 7, 9, and 11 weeks post infection. Blood samples for sera from five naive mice were used as controls.

To determine IgG levels induced in the *S. japonicum*-infected BALB/c mice, ELISA plate was coated with recombinant SjILP (1 μg/mL, 100 μL/well) in coating buffer overnight at 4 °C, followed by blocking with blocking buffer (1% BSA in PBST) at 37 °C, 1 h. Serum samples (1:100 in blocking buffer, 100 μL/well) were added to wells and incubated at 37 °C, 1 h. A biotin-SP-conjugated goat anti-mouse IgG (Fc specific)-biotin antibody (Jackson ImmunoResearch, PA, USA) was added as a secondary antibody (1:10,000, 100 μL/well) and samples were incubated at 37 °C, 1 h. Streptavidin-HRP (BD Pharmingen, CA, USA) (1:10,000) was then applied in 100 μL/well. Phosphate buffered saline with Tween 20 (PBST) washes were applied (5 times × 2 min) after each step. Reactions were developed using a 3,3′,5,5′-tetramethylbenzidine (TMB) substrate (100 μL/well) for 5 min and stopped using 2M sodium hydroxide (50 μL/well). Optical density (OD) values were read at 450 nm, and all tests were run in duplicate. A positive antibody response was defined as an OD450 higher than 2.1 times the mean of OD450 of the serum samples from control mice.

### 4.7. Statistical Analysis

Data are presented as the mean ± SE. Group comparisons were assessed by one-way ANOVA or *t*-test for statistically significant differences, defined as a *p* value ≤ 0.05 using GraphPad Prism software (Version 7.02). (* *p* value ≤ 0.05; ** *p* value ≤ 0.001; *** *p* value ≤ 0.0001).

## 5. Conclusions

In summary, this report sheds new light on the differential expression of SjILP in different developmental stages of *S. japonicum*, findings that add to its recognised role in binding parasite IRs and activating the schistosome insulin signaling pathway in the mammalian host, functions critical for parasite survival.
